# MARCH to combat Zika virus infection

**DOI:** 10.1038/s41423-025-01359-7

**Published:** 2025-11-28

**Authors:** Upendra Pradeep Lambe, Rohit K. Jangra

**Affiliations:** Department of Microbiology and Immunology, Center for Applied Immunology and Pathological Processes, Louisiana State University Health Sciences Center-Shreveport, Shreveport, Louisiana 71103 USA

**Keywords:** Viral infection, Proteolysis

**Fig. 1:**
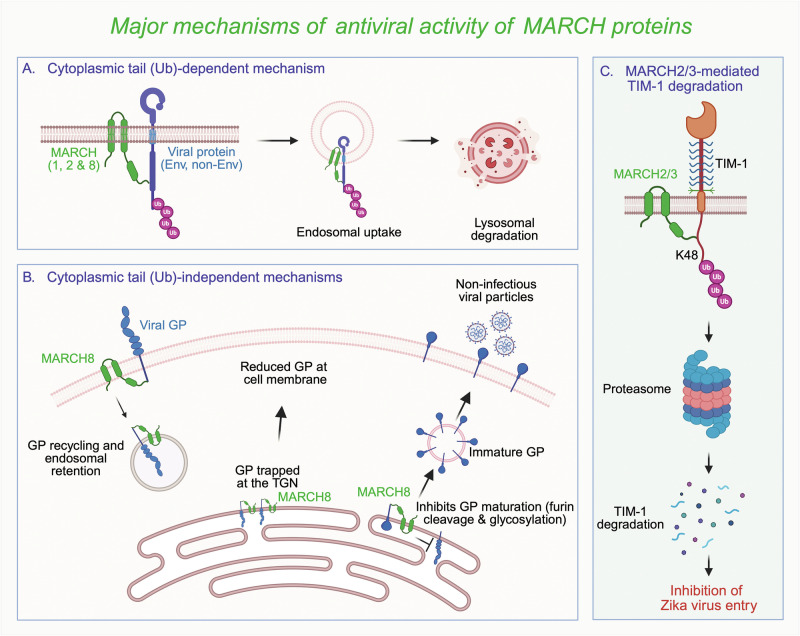
Major mechanisms of the antiviral activity of MARCH proteins. MARCH proteins typically exert their antiviral activity by downregulating viral proteins. **A** MARCH1/2/8 can mediate the polyubiquitination of lysine residues in the cytoplasmic tails of both envelope and nonenvelope viral proteins to target them for lysosomal degradation. **B** The MARCH8 protein can also downregulate proteins independently of ubiquitin and the cytoplasmic tail by retaining them in endosomes and the trans-Golgi network (TGN), as well as by inhibiting glycoprotein maturation through blockade of glycosylation and cleavage by the furin enzyme. **C** An elegant study by Zhang et al. revealed a new mechanism by which MARCH2/3 proteins restrict Zika virus (ZIKV) infection by reducing the expression of the TIM-1 protein, the cellular receptor for ZIKV, through its polyubiquitination followed by proteasomal degradation. Env envelope proteins, non-Env nonenvelope proteins, Ub ubiquitin, GP glycoprotein, TIM-1 T-cell immunoglobulin and mucin domain-1, TGN trans-Golgi network. Image created in https://BioRender.com

The ubiquitination of proteins, followed by their degradation via the proteasome or autophagosome, is a key mechanism for the posttranslational regulation of proteins in cells. The specificity of this process is primarily dictated by the E3 ubiquitin ligases, which are classified into three main types: the really interesting new gene (RING), the RING-between-RING (RBR), and the homologous to the human papillomavirus E6 protein-associated protein (E6-AP) carboxyl terminus (HECT). Among the RING-type E3 ubiquitin ligases are the membrane-associated or membrane-proximal RING-CH (MARCH) proteins, which regulate the trafficking and levels of cellular and viral proteins, including immune receptors, innate immune response proteins, and viral glycoproteins [1]. Eleven MARCH proteins, named MARCH1-11, are encoded in the human genome, and some of them (primarily MARCH8 along with MARCH1 and MARCH2) have been implicated in antiviral defense against RNA viruses such as human immunodeficiency virus (HIV-1), influenza virus, Ebola virus, SARS-CoV-2, and respiratory syncytial virus (RSV) [2]. MARCH proteins usually exert their antiviral effects by decreasing the incorporation of viral glycoproteins into virions through ubiquitin- and cytoplasmic tail-dependent lysosomal degradation or ubiquitin- and cytoplasmic tail-independent retention in endosomes and the trans-Golgi network and/or blockade of glycoprotein maturation (Fig. 1). MARCH proteins can also restrict virus infection by selectively degrading nonenvelope viral proteins. Zhang et al. described a novel way by which MARCH proteins can inhibit virus infection: by downregulating the host receptor T-cell immunoglobulin and mucin domain-1 (TIM-1), which facilitates Zika virus (ZIKV) entry, to block infection (Fig. 1) [3].

To identify the MARCH protein(s) capable of downregulating TIM-1 expression, the authors coexpressed all 11 MARCH proteins, each with TIM-1, in human cells. MARCH2 and MARCH3 proteins significantly reduce TIM-1 levels. By knocking out MARCH2 and MARCH3 separately or together in mice, they demonstrated that MARCH2 and MARCH3 function as negative regulators of TIM-1 under physiological conditions. Although they could each downregulate TIM-1 separately, double knockout mice presented a much greater reduction in TIM-1 levels, highlighting their redundant roles, which makes sense given their close phylogenetic relationship and cellular localization. Coimmunoprecipitation experiments revealed that both MARCH2 and MARCH3 interact with the TIM-1 receptor to promote K48-linked polyubiquitination of TIM-1. Detailed mutagenesis experiments revealed that MARCH2 causes polyubiquitination at lysine 338 of TIM-1, whereas MARCH3 targets lysine 346, leading to proteasomal degradation of TIM-1. Complementary overexpression experiments verified the specific involvement of MARCH2/3 in TIM-1 degradation. In contrast, MARCH2 and MARCH3 did not have any downregulatory effects on the other cellular receptor, CXCR7. Therefore, MARCH2/3 control the steady-state levels of TIM-1 both in cell culture and in vivo.

Cell culture experiments demonstrated that MARCH2/3-mediated downregulation of TIM-1 decreased ZIKV infection. Knocking out either MARCH2 or MARCH3 increased TIM-1 levels, resulting in a moderate increase in ZIKV infection. Furthermore, knocking out both MARCH2 and MARCH3 strongly reduced ZIKV infection in wild-type cells, but this effect was not detected in TIM-1 knockout cells. This pattern was consistently observed across various cell lines, including hepatic (Huh7), placental (BeWo), and neuronal (U87, U251) cells. Dengue virus (DENV), another flavivirus that uses the TIM-1 receptor, infection was also increased in MARCH2/3 double-knockout cells. However, there was no effect on influenza H3N2 or Sendai virus, which do not depend on TIM-1 for entry. Finally, the introduction of a ubiquitination-resistant TIM-1 mutant (K338R/K346R) into TIM-1 knockout cells resulted in greater ZIKV infectivity than that in cells complemented with wild-type TIM-1, confirming that the ubiquitin-ligase activity of MARCH2/3 is involved in TIM-1 degradation and ZIKV infectivity.

To validate these findings in an animal model, the authors infected MARCH2, MARCH3, and MARCH2/3 double knockout animals with ZIKV. These knockout mice presented progressively increased TIM-1 levels in their tissues. Notably, compared with wild-type infection, ZIKV infection in MARCH2/3 knockout mice caused significant weight loss and increased viral loads in the spleen, liver, and lung tissues. This effect was especially pronounced in the MARCH2/3 double-knockout mice. Compared with those in humans, the MARCH2 and MARCH3 ligases recognize different lysine residues in mice, inducing the polyubiquitination of TIM-1 at K265 and K276, respectively. As a result, reconstituting the mouse TIM-1 mutant (K265R/K267R), which is resistant to MARCH2/3-mediated polyubiquitination, led to increased ZIKV infection and replication compared with reconstitution with wild-type TIM-1. Collectively, these findings support the conclusion that MARCH2 and MARCH3 restrict ZIKV infection by downregulating the expression of the viral receptor TIM-1. On the basis of these results, future research could explore whether enhancing MARCH2/3 activity or inhibiting the TIM-1 receptor might reduce infections caused by other viruses, such as filoviruses, dengue virus, and chikungunya virus, which rely on TIM-1 for entry [4, 5].

This elegant study also raises many intriguing questions. Where does TIM-1 ubiquitination by MARCH2/3 occur: in the ER or at the plasma membrane? Is the PDZ-binding domain of MARCH2/3 essential for their TIM-1 downregulation activity, considering that mutations in this domain cause ER retention of MARCH2/3 proteins [6]? Experiments with mutants of the PDZ-binding domain may help clarify this.

Among the 11 MARCH proteins, why do only MARCH2 and 3 downregulate TIM-1 while the others do not? What determines the substrate specificity of MARCH proteins? What, if any, is the evolutionary advantage of redundancy between MARCH2 and MARCH3 in TIM-1 regulation? Viruses are experts in countering host defense mechanisms. However, it is unknown whether ZIKV or other TIM-1-dependent viruses encode ways to bypass MARCH2/3 protein-mediated downregulation.

How are the levels of MARCH2/3 regulated at both the transcriptional and posttranslational stages? Interestingly, MARCH2 is upregulated by HIV-1 infection [7]. It would be interesting to evaluate whether MARCH2/3 expression is induced by TIM-1-dependent viruses. Furthermore, each of the human MARCH2/3 proteins has two isoforms. It is unknown whether they differ in their capacity to downregulate TIM-1.

MARCH1, 2, and 8 proteins can also downregulate transferrin receptor (TfR) [8]. This protein is essential for many virus infections, including those caused by deadly arenaviruses [9]. While we are still far from developing medical interventions targeting MARCH2/3 to limit virus infections, this research by Zhang et al. undoubtedly opens new avenues for potential targets against many human pathogens. However, for any therapeutic application in which MARCH proteins are targeted, the kinetics of TIM-1 and TfR downregulation and control of viral infections need to be clarified.

In contrast, some MARCH proteins, such as MARCH8, can downregulate cyclic GMP-AMP synthase (cGAS)-mediated innate immune responses to induce a proviral response during herpes simplex virus (HSV-1) infection [10]. MARCH8 also has a proviral role in hepatitis C virus, dengue virus, and Zika virus infections [2]. Therefore, teasing out the antiviral and proviral activities of MARCH proteins will be really interesting.
